# A new procedure for determining the genetic basis of a physiological process in a non-model species, illustrated by cold induced angiogenesis in the carp

**DOI:** 10.1186/1471-2164-10-490

**Published:** 2009-10-23

**Authors:** John MJ Herbert, Francesca M Buffa, Henrik Vorschmitt, Stuart Egginton, Roy Bicknell

**Affiliations:** 1 Cancer Research UK Angiogenesis Group, Institute for Biomedical Research, Schools of Immunity and Infection and Cancer studies, College of Medicine and Dentistry, University of Birmingham, Birmingham, B15 2TT, UK; 2 Cancer Research UK, Weatherall Institute of Molecular Medicine, University of Oxford, John Radcliffe Hospital, Oxford, OX3 9DS, UK; 3 Division of Medical Sciences, Medical School, University of Birmingham, Edgbaston, Birmingham, B15 2TT, UK

## Abstract

**Background:**

Physiological processes occur in many species for which there is yet no sequenced genome and for which we would like to identify the genetic basis. For example, some species increase their vascular network to minimise the effects of reduced oxygen diffusion and increased blood viscosity associated with low temperatures. Since many angiogenic and endothelial genes have been discovered in man, functional homolog relationships between carp, zebrafish and human were used to predict the genetic basis of cold-induced angiogenesis in *Cyprinus Carpio *(carp). In this work, carp sequences were collected and built into contigs. Human-carp functional homolog relationships were derived via zebrafish using a new Conditional Stepped Reciprocal Best Hit (CSRBH) protocol. Data sources including publications, Gene Ontology and cDNA libraries were then used to predict the identity of known or potential angiogenic genes. Finally, re-analyses of cold carp microarray data identified carp genes up-regulated in response to low temperatures in heart and muscle.

**Results:**

The CSRBH approach outperformed all other methods and attained 8,726 carp to human functional homolog relationships for 16,650 contiguous sequences. This represented 3,762 non-redundant genes and 908 of them were predicted to have a role in angiogenesis. The total number of up-regulated differentially expressed genes was 698 and 171 of them were putatively angiogenic. Of these, 5 genes representing the functional homologs NCL, RHOA, MMP9, GRN and MAPK1 are angiogenesis-related genes expressed in response to low temperature.

**Conclusion:**

We show that CSRBH functional homologs relationships and re-analyses of gene expression data can be combined in a non-model species to predict genes of biological interest before a genome sequence is fully available. Programs to run these analyses locally are available from .

## Background

### The cold-induced angiogenic response

Angiogenesis can be initiated by a variety of stimuli. For example, angiogenesis is induced by airway smooth muscle strain in chronic asthmatics [1], by exercise [2-6] and in some species by cold exposure [7-10]. Many species are subjected to annual cycles of environmental cooling that represents a significant challenge for the cardiovascular system due to the effects of reduced oxygen diffusion and increased blood viscosity associated with low temperatures [7,11,12]. One way of overcoming limitations to aerobic activity is to increase tissue capillary supply. Although the underlying process is unclear, cold-induced angiogenesis may respond to altered hormonal levels or changes in the mechanical environment of endothelial cells (ECs) [13]. Even within the mammalian literature, it is apparent that a number of types of capillary growth exist, e.g. pathological vs. physiological angiogenesis, albeit under the influence of a restricted set of genes [14].

In order to improve the signal to noise ratio from the wealth of published data and reduce the influence of host tissue in the readout, gene expression profiles for endothelial-specific up-regulated genes have recently been identified [15,16]. No such information exists for fish, although their use as an experimental model is increasing due to the additional interventions not possible with mammals [17-22]. For the first time, we have used an *in silico *approach to identify whether orthologs between man and fish could be identified that were associated with angiogenesis, then used re-analysis of microarray data to see if these genes were differentially expressed on cold exposure and finally we determined what proportion of the up-regulated genes were angiogenic or EC-specific. We anticipate this approach could be useful for similar studies in non-model species for which a genome sequence is not available but the human orthologs involved in the biological process are known.

## Results and Discussion

### Gene assignment by ortholog identification

Orthologs (functional homologs), as a whole, perform an equivalent function in respective genomes [23-25]. Based on this assumption, carp orthologs of known angiogenic human genes provide genes pivotal in their cold angiogenic response. Cold carp data from a study by Gracey et al. 2004 [26] was to be used in this work to predict the identity of cold induced angiogenic genes. The genes assigned to carp sequences from the original article [26] were not applicable to this study as they used a BLASTX against multiple species databases and only looked for the best hit without using a control step. In addition, the databases searched were from 2004, several years out of date, and a more stringent method of sequence contig construction was used in this work (see methods).

A problem to be overcome was how best to assign human orthologs to 19,995 anonymous EST carp sequences collected from Genbank and CarpBASE (additional file 1). Many of these sequences were less than 500 bases and many of them contain partial or complete untranslated regions. An obvious first step was to cluster them, using CAP3 [27], to extend the sequence length and improve the quality of any subsequent analyses. This reduced the sequence count to a total of 16,650 sequence contigs and singletons (additional file 2). The next challenge was to identify their human orthologs.

### Choosing the ortholog assignment method

Homologs, in the strictest sense, are characters that have been passed down following a speciation event from a common ancestor [25,28]. A gene in two species derived from a single gene in their common ancestor is defined as an ortholog (functional homolog) and usually has the same or similar function between species [25,28], i.e. the same gene in different organisms are orthologs [29]. Conversely, paralogs are defined as genes derived from a single gene sequence duplication event and usually have different functions [25,28,30]. Orthologs and paralogs are not the only gene histories and several other events can take place, see [28] for a full list. In this work, the sole aim was to find the most likely functional human homologs of non-model transcripts and did not attempt to define or classify paralogs or any other forms of gene histories.

Many bioinformatic programs and pre-computed databases of orthologs exist, a number of which are listed in a recent review [28]. Essentially, there are 3 methods of finding orthologs, tree based, graph based and a combination of the two. Tree methods use sequence alignments and phylogenetic trees for predicting evolutionary relationships, while graph based methods use pairwise sequence similarity search methods (such as BLAST) to predict orthologs. The review highlights the merits of each program or database and it provides the reader with a flow chart (their figure four) to assist in the best choice of tool for a particular application. After investigation of the recommended programs it was decided that none were applicable to the high-throughput analysis needed to cope with the > 16 K incomplete anonymous carp nucleotide sequences. The main reasons were the inability of these programs to be queried with a high volume of data through their web servers or the databases were several years out of date.

Further literature searches revealed a tool called BLAST on orthologous groups (BLASTO) [31] which searches a query sequence against a database of orthologous groups (a collection of homologous genes from at least two genomes [28]) as a single unit. Although this tool enabled searches against several combination method pre-computed databases including Homologene [32,33], it could only deal with one sequence at a time. Additionally, the BLASTO form did not permit the user to change BLAST filtering options which, as seen below, makes a difference to the quality and quantity of successful ortholog predictions.

### Ortholog assignments, RBH and filtering options

A graph based, nearest neighbour [28] approach, RBH, was considered the best option since constructing multiple sequence alignments and phylogenetic trees from incomplete nucleotide sequences would be inaccurate and not amenable to high throughput. Strengthening this decision, a performance assessment of different ortholog prediction methods by Altenhoff and Dessimoz 2009 [34] found the RBH performed well in comparison with the other methods.

**Figure 1:**
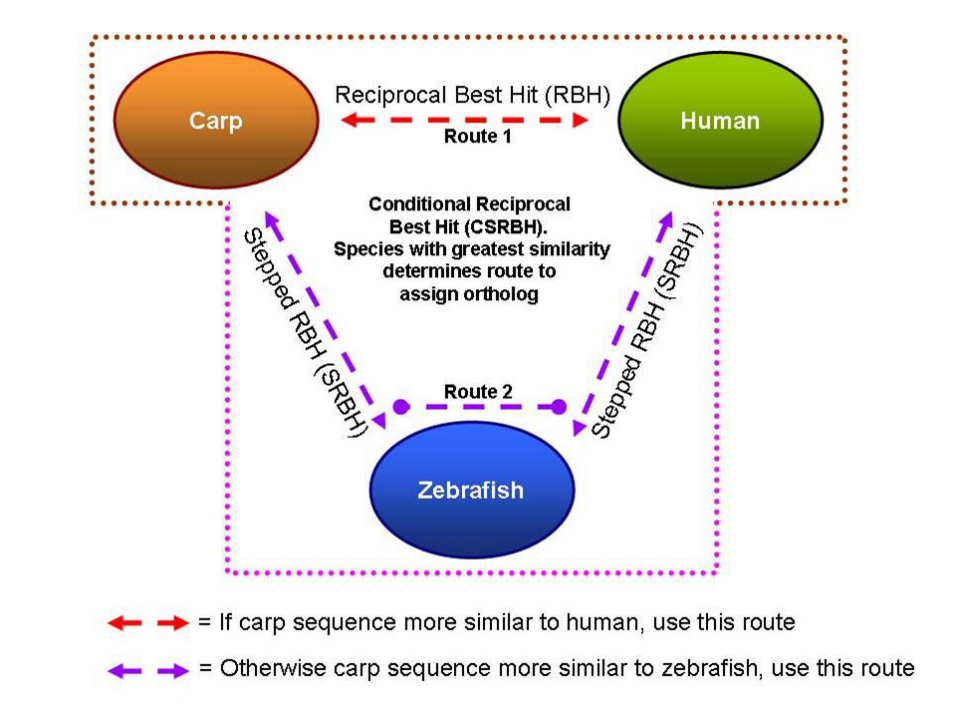
**This figure shows the routes to ortholog assignment**. Reciprocal Best Hit (RBH), labelled route 1, conveys if two genes in carp and human find each other as the best hit in the other species. Stepped Reciprocal Best Hit, SRBH (route 2), extends RBH by using a closely related species, zebrafish, to enhance ortholog assignments. This is possible as the zebrafish has many more sequences in the databases, its genome is significantly more annotated and it has a genome sequence available. The Conditional Stepped Reciprocal Best Hit combines both RBH and SRBH as some sequences are still missing from zebrafish.

Reciprocal Best Hit (RBH) [28,29] is a technique using BLAST to first search a query sequence against a genome. If the resulting best hit matches the original query sequence from a reciprocal BLAST then it is an ortholog termed RBH. A diagrammatical representation of the RBH process of carp vs. human is shown in figure 1, labelled route 1.

**Figure 2:**
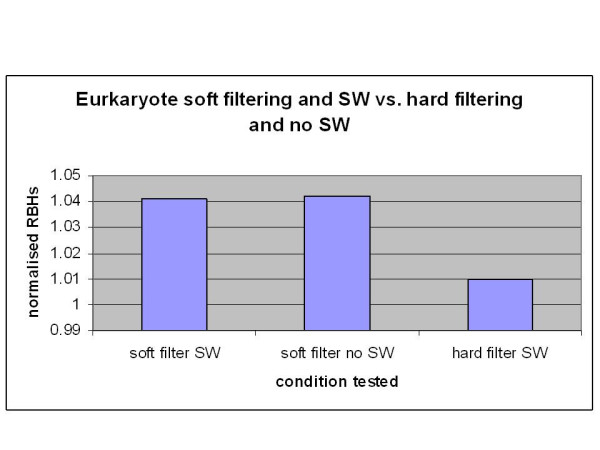
**This figure summarises the number of successful RBHs found using different BLAST options**. It can be seen that soft filtering (-F "m S") makes a significant difference to the number of successful RBHs attained. The Smith-Waterman algorithm, on the other hand, made little difference to the number of successful RBHs. The results of each filtering option were normalised to the default options of BLAST (-F T -s F).

An article by Moreno-Hagelsieb and Latimer 2008 [29] tested ortholog RBH searching using different BLAST options. The tests utilised bacterial genomes, neighbouring orthologs and paralogs to estimate error rates of ortholog assignments by RBH. They found the soft filtering option -F "m S" with the addition of Smith-Waterman (SW) alignments (-s T) were optimal for RBH success. These options were tested for eukaryotes using carp vs. human RBH searches. Four different BLAST options were tested: soft filtering (-F "m S") with and without SW (-s T) and hard filtering with and without SW. The results revealed very little difference in the number of successful RBH with or without the SW algorithm against their findings of 10%. In contrast, as with prokaryotes, soft filtering increased the number of RBHs by 2% (209 RBHs). Table 1 shows the number of successful orthologs found using carp *vs*. human RBH for the different filtering options. Figure 2 shows a bar chart of the successful RBH results normalised to the default BLAST options (-F T, -s F).

**Table 1:** The effect of BLAST filtering options on the performance of RBH

**Condition**	**BLAST options**	**Number of RBHs**	**Normalized value**
Soft filtering with SW	-F "m S" -s T	6773	1.04

Soft filtering no SW	-F "m S" -s F	6779	1.04

Hard filtering with SW	-F T -s T	6570	1.01

Hard filtering with no SW	-F T -s F	6507	1

This table shows the performance of RBH using the different BLAST filtering options with eukaryotic sequences. The results from this analysis showed the soft filtering option with no Smith and Waterman produced the highest number of successful RBHs.

Contrary to the findings of Moreno-Hagelsieb and Latimer 2008 [29], who showed the SW algorithm improved results by 10%, in this study using SW produced 0.0004% (6) less successful results. However the analyses in this work were slightly different to that of Moreno-Hagelsieb and Latimer and are listed here:

1) They used complete bacterial genomes: A) These sequences are generated from genomic DNA and are sequenced in both strands. B) The Carp sequences are generated from single pass (one strand) cDNA sequencing from RNA and, as such, are likely to contain more errors.

2) The protein sequences used in assessing RBH success: A) Moreno-Hagelsieb and Latimer used full length and defined proteins from complete bacterial genomes. B) The carp sequences were partial proteins from 6 frame nucleotide translations and from a eukaryotic and not a prokaryotic species.

3) The algorithm options used with BLAST were not exactly the same. A) Moreno-Hagelsieb and Latimer used the blastpgp algorithm from the NCBI [35] BLAST, which has the Smith-Waterman -s T option available. B) In this work blastall using blastx (carp vs. human) and tblastn (human vs. carp) were used. The -s T option is only available for tblastn and not blastx with NCBI blastall. Note: blastpgp can only be used on protein sequences and not nucleotides.

4) Some cDNAs span genes on different chromosome strands and is another way RBH results could be affected (E.g. cDNA DY691296).

However, even with these differences, the results were very surprising because of other research [36] and considering the -s F option is a fast approximation of the SW algorithm (see methods for details). One thing to bear in mind is an e-value of 1e-5 and soft filtering were employed in this work, which in itself, would remove many spurious matches otherwise found using the default BLAST heuristic. A further investigation into this revealed that the genes successfully found with each method did not totally overlap. The heuristic BLAST alignment method predicted 62 functional homologs with RBH that SW did not find and the SW alignment method predicted 63 that failed with the BLAST heuristic. The intersection of results totally concurred. The questions to answer here were; why did one algorithm fail RBH, whilst the other method was successful? And of these differences, which algorithm produced the more believably evolutionary related RBH successes?

To investigate this finding further and to decide on which method to use in this work, a manual inspection of sequence alignments was needed in a systematic way to avoid the problem of biased cherry picking. This was done using a random number generator to pick 12 sequences to investigate (see methods), 6 from each algorithm RBH success. So the investigation was to see if the results were believable for each algorithm. The results found that ~50% (additional file 3) of results suggested the default BLAST option found the more likely evolutionary related sequence as it found higher similar matches but at shorter alignment lengths, which is believable as SW is good at finding distant homology. However, this was not always the case and the different sequence alignment algorithms were correct for different sequences. As a consequence it was not possible to automate the choice without manually viewing the results. Therefore, in this work the results generated using the default BLAST heuristic was used for the final data. However, it is recommended for other researchers using these methods on small datasets to use the intersection of both methods or the SW alone (based on previous research [36]). This is not a problem for small numbers of sequences but for Next Generation Sequence data, for example, it is recommended the default heuristic be used as it will take weeks to compute SW alignments.

All but one cold induced angiogenic gene were found with both algorithms and users of this data can be confident in the ortholog assignments of these genes. The exception was the plasminogen gene PLG (cDNA CA965299).

### Taking advantage of a closely related vertebrate

Zebrafish are a model teleost organism used in the elucidation of vertebrate development, molecular, genetic, genomic and evolutionary biology [37]. As such its genome has been sequenced with extensive annotation and currently in the sequence databases there are over 6 million ESTs and 28 thousand Refseq proteins. Carp, on the other hand, currently has ~20 thousand ESTs and no genome sequence. It was possible to take advantage of this annotation because of the close evolutionary relationship carp has to zebrafish in that they diverged from their last common ancestor only 50 million years ago [38]. Previous groups have shown that close genome evolutionary relationships can aid ortholog identification [39,40]. In the present study, zebrafish was also utilised as a stepping stone approach to ortholog identification. Figure 1, labelled route 2, shows a Stepped Reciprocal Best Hit (SRBH) approach where RBH analysis is carried out twice, once between carp and zebrafish and then between zebrafish and human. If both were successful, then a carp-human ortholog was assigned. Using the SRBH analysis gained a further 8% (8,145 sequences) successfully assigned orthologs.

### Teleost genome duplication and SRBH

Although there is a discrepancy as to when it happened, there is much evidence for a genome duplication event in teleosts [22,41-47]. A good example in zebrafish is the paralogs Ets1 and Etsrp which both lie next to fli1a and fli1b, respectively, but on different chromosomes. These genes are important for vasculogenesis and angiogenesis [48,49]. From a SRBH analysis, zebrafish genes ets1 and etsrp best match the human gene ETS1 and fli1a, and fli1b best matches the human FLI1, suggesting a genome duplication event occurred in zebrafish. Because of duplicate genes, the best and second best hits to zebrafish and carp genes were used to attain a successful SRBH. The impact of genome duplication in ortholog searching can be seen in the following example. The zebrafish peptide NP_958883 was the best hit to CA969258. However, the peptide NP_958883 best matched human NP_003002. Blasting NP_003002 back against zebrafish revealed two very similar sequences, NP_958876 and NP_958883. NP_958876 was the best hit and therefore failed SRBH (additional file 4). This is due to genome duplication of the zebrafish genome and thus both genes in zebrafish can be considered orthologs to the same human gene.

### RBH and a comparison with Homologene

Homologene is a database of orthologous groups defined with BLAST pairwise alignments and phylogenetic trees [32,33]. Since CSRBH included a zebrafish and human ortholog RBH analysis, a comparison was made between Homologene and this RBH step to see if the zebrafish to human ortholog assignments from this work agreed. From the 8,145 successful RBHs, only 7,020 could be compared as only these had both human and zebrafish genes present in the Homologene data. This fact alone gives support to using this approach as a user supplies all genes, and as such, all are present. From these ortholog assignments, 85% agreed with Homologene orthologous groups. A manual investigation of some of those that disagreed suggests Homologene does not always perform well for a proportion of duplicated genes. For instance, one example, the zebrafish actc1l gene best matched the human ACTC1 gene with 99% identity over the full alignment length of the gene but Homologene put actc1l in the ACTB orthologous group (id: 110648) where alignment percent identity was less at 94% (additional file 5). The full results of the comparison can be viewed in additional file 6. In addition, ten alignments were chosen randomly and manually investigated (additional file 7) to see which source of data was most realistic. The results of this suggested 8 out of 10 ortholog assignments were correct with RBH whilst Homologene put the genes into different orthologous groups.

### A full comparison of RBH with Homologene

A full analysis was also performed that took all human proteins in the Refseq database of proteins and RBH searched against the full database of zebrafish Refseq proteins. On July 22nd 2009, there were 29,428 proteins in the human database. 19,648 of these proteins resulted in a successful RBH analysis against zebrafish. Build 64 of Homologene (16th July 2009) contained 19,571 human proteins. Therefore 33% of the human proteome was not represented in Homologene at this time and only 12,035 of the 19,571 human proteins had a zebrafish homolog protein in the same orthologous group.

There were 653 human/zebrafish orthologs identified by Homologene only and not with the RBH procedure. In contrast, there were 8,266 human/zebrafish orthologs found using the RBH but not with in Homologene. 11,382 human proteins were found in both Homologene and in the RBH analysis. 10,099 of these agreed, an 89% agreement. This is very similar to the result above with only the carp data.

These findings, particularly the fact that Homologene only had 12,035 human proteins with a zebrafish ortholog and the findings of Altenhoff [34], give a user some further support and confidence to using a RBH ortholog assignment approach rather than phylogenetic tree methods for analysing large amounts of data from a non-model species in a quick and fairly accurate manner.

The SRBH approach is endorsed by the fact other researchers have used closely related species to predict genes and sequence motifs [39,40]. However, it should be noted that the comparisons done here with Homologene only validates the RBH method but not the CSRBH as, to definitively validate the CSRBH approach, the carp genome and annotation need to be more complete.

In addition, the CSRBH approach does not attempt to better or replace the efforts of databases such as Homologene. One reason is that it does not attempt to deal with paralogs and other sources of gene histories but only with the most functional homologs (orthologs), though some attention was given to the teleost genome duplication event. So there are examples where Homologene better assigns orthologs than the methods used here. Having said that, with a non-model species not present in Homologene, this approach is excellent at giving a good approximation of the human functional homologs of researchers' genes.

### CSRBH, combining RBH and SRBH to find further orthologs

Although adding the zebrafish intermediary step (SRBH) improved ortholog assignment by 8%, 581 assignments that failed using this procedure were successfully found using a carp vs. human RBH alone (figure 1, route 1). Inspection of a random sample of these found two examples of why this occurred. BLAST of carp EST EC394432 vs. zebrafish best hit the gene smyhc3 and the reciprocal BLAST vs. carp found EST AB231800 as the best hit. However, smyhc3 was the fifth best hit to AB231800, thus failing the SRBH analysis (additional file 8). However, this gene is successfully assigned a human ortholog from an RBH analysis (figure 1, route 1).

Even more compelling was the situation with EST AU052068 (additional file 9). It aligns with the human protein NP_003745 as the best hit and the alignment was highly similar with a percent identity of 91% over 200 amino acids. The same EST best aligned to the zebrafish protein NP_956083 with 37% identity over 200 amino acids. Hence, the EST is significantly more similar to the human protein rather than the zebrafish. The human protein NP_003745 was BLAST searched against zebrafish to see if the full-length protein found a likely ortholog in zebrafish. The best hit to the human protein was NP_956083, the same as found by searching EST AU052068. Therefore, there is evidence that either some genes do not exist in zebrafish and do so in carp or are not currently found in zebrafish as the genome is not yet completely sequenced or annotated. Another piece of evidence is, if a simple BLAST search is performed against the multiple species NR database of proteins at the NCBI [35], 24% of carp sequences preferentially match a human protein, whilst 30% match a zebrafish protein. This supports the use of a Conditional Stepped Reciprocal Best Hit (CSRBH) approach, which uses whichever species is a closer match to the query and performs the appropriate ortholog prediction route (figure 1). In addition, for any that fail, a rescue was be performed by searching through the other route.

**Figure 3:**
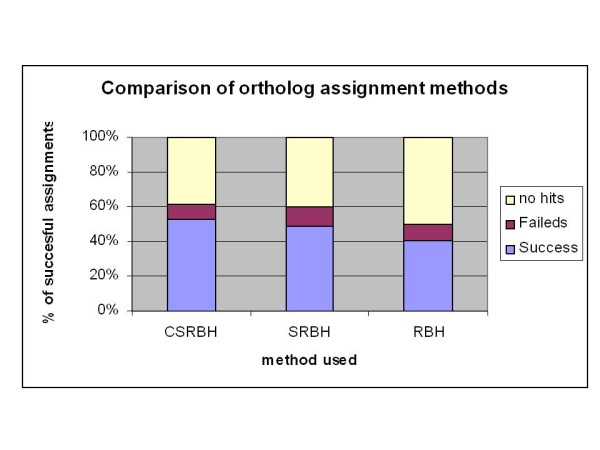
**This figure clarifies which of the methods was optimal for finding human-carp orthologs**. CSRBH performed best with 52% of contiguous sequences successfully assigned an ortholog as against 49% and 41% for SRBH and RBH respectively.

CSRBH outperformed all other methods and produced 3% and 11% more successful ortholog assignments than the SRBH and RBH, respectively. In total 8,726 contigs were assigned an ortholog, totalling 3,762 non-redundant genes (see additional file 10). See table 2 and figure 3 for the number of successful ortholog assignments for all the different analysis methods used in this work. We propose that CSRBH should be the method of choice for researchers working with non-model species which have no sequenced genome and partially sequenced genes.

**Table 2:** A summary of the results for the bioinformatic analyses

**Number of contiguous sequences**	**RBH successful orthologs**	**SRBH successful orthologs**	**CSRBH successful orthologs**	**Number of genes**	**Number angiogenic genes**	**Number of up-regulated genes**	**Number of up-regulated angiogenic genes**
16650	6779	8145	8726	3762	908	567	135

This table displays the number of human protein orthologs found using CSRBH for the 16,650 cDNA carp contiguous sequences. 3,762 total none redundant genes were found and 908 of them were predicted as angiogenesis genes. From the re-analysis, 567 genes were up-regulated and 24% of them were predicted as angiogenesis genes.

A weakness with this approach is that it does not determine an orthologs absolute gene history and it will not report to a user whether the ortholog found was derived from a gene duplication event or from a speciation. That said, these methods will enable a user to quickly find the most likely functional homolog of a gene.

### Prediction of angiogenic genes

#### Established angiogenic genes

Previous groups have developed complex data mining methods using decision trees to identify genes of interest [50]. In this study, 4 simple strategies were combined to generate as a complete a set of angiogenic genes as possible. First the cardiovascular and angiogenesis groups at Birmingham University used their knowledge to produce a list of angiogenic genes that included those from the recent literature [51] and the SABiosciences GEArray^® ^Human Angiogenesis Microarray [52]. A total of 260 angiogenesis related genes were predicted this way and a list of 73 genes were contained in the successful CSRBH results (additional file 11, column D, labelled in house). However, it is possible many other genes have an undiscovered role in angiogenesis and, therefore, three further methods were employed to predict additional angiogenic genes.

#### Putative angiogenic genes

The second and third approaches used Perl programs to scan article abstracts from PubMed [53] and Gene Ontology processes from AMIGO [54] using the following angiogenic related keywords as baits: 'angiogenic, angiogenesis, neovascularization, neovascularisation, vasculogenesis, hypoxia, endoth VEGF'. This predicted a further 737 angiogenic genes (additional file 11, columns E and F respectively).

A fourth method used gene expression of ECs. During the activation of angiogenesis, ECs become activated and switch on several signalling pathways that cause proliferation, migration and invasion [55,56]. This angiogenic phenotype leads to an up-regulation of EC genes. Therefore, genes up-regulated in the endothelial transcriptome could be angiogenic. The endothelium transcriptome has been extensively defined [15,16] and genes up-regulated in ECs were compared with the human/carp orthologs to identify additional putative angiogenic genes; 98 were found (additional file 11, columns G and H).

#### Total angiogenic genes

Combining the results from the four data mining methods gave a total of 908 non-redundant predicted angiogenic genes (additional file 11). We postulate this gave us a comprehensive set and the best chance of discovering cold-induced angiogenic genes from the carp data. However, it should be noted, and the authors fully acknowledge, that not every gene predicted here is a genuine angiogenesis gene and there will be some false positives. Very few bioinformatic predictions are 100% foolproof and this study is aimed at guiding the bench scientist into making more informed decisions before going into the laboratory. The number of different methods that predicted a gene angiogenic now ranks the genes. For example, the gene HIF1A is a well-known angiogenic gene and was predicted as such by all four methods. Column I entitled "Number of methods" in additional file 11 gives the number of methods predicting a gene angiogenic and is ranked in a descending order. Conversely, genes that are not so well known to be angiogenic genes are only found by one or two methods. If this approach is employed and genes selected that were predicted by more than one method, reduces the high percentage of angiogenic genes from 25% down to 9. This is reduced further if you only take genes with 3 and/or 4 methods. But given the full list, a researcher can decide based on the presence of functional domains or personal knowledge which ones are of interest to investigate with full knowledge that the human functional homolog was only loosely associated with the biological process of interest.

### Re-analysis of gene expression data

Three established methods of measuring gene expression used in biological research are microarrays, cDNA and Serial Analysis of Gene Expression (SAGE) libraries. To investigate numerous pathologies many institutes around the world have generated, analysed and deposited large amounts of expression data into public repositories [57]. Examples of such databases are the Gene Expression Omnibus (GEO), Cancer Genome Anatomy Project (CGAP), National Center for Biotechnology Information (NCBI) and European Bioinformatics Institute (EBI) arrayExpress. It has been shown that data mining of relevant data sets can successfully lead to the identification of biologically interesting targets [15,16,58-67].

### Cold response differentially expressed genes

Carp is an ectotherm with a body temperature similar to the water it is immersed in and can adapt to a range of temperatures (eurythermal). A recent study investigated physiological adaptation of carp exposed to gradually lower temperatures [26]. Microarray analysis measured the change in gene expression, in several tissues, at several cooling stages from 30 to 10°C *vs*. a control temperature of 30°C. Overall, 252 transcriptional regulatory, RNA splicing and translation control genes were found to be up-regulated in all tissues. Interestingly, some tissue-specific affects were also seen, e.g. in the glycolytic pathway of brain and lipid metabolism of liver. This data set proved appropriate for re-analysis to predict cold-induced angiogenic genes in skeletal and heart muscle tissues.

### Persistently up-regulated genes

Two strategies were applied to the data. The first utilised the data without intervention for heart and muscle tissues, employing a threshold score > = 2. The total number of up-regulated cDNAs for heart was 748, 270 non-redundant genes, and for muscle 1230 cDNAs, 475 genes. Any cDNAs with significant contradicting expression were removed. For both heart and muscle combined there were 589 non-redundant genes, 143 of which were predicted angiogenic genes (see table 3 and additional file 12; columns c and d for both heart and muscle sheets).

**Table 3:** A numerical breakdown of cDNAs differentially expressed due to cold temperature

	**Heart tissue**	**Muscle tissue**	**Combined results**
**Persistent cDNAs**	748	1230	1603

**Persistent genes**	270	475	589

**Persistent angiogenic genes**	73	102	143

**Ratio cDNAs**	3	82	83

**Ratio genes**	1	29	30

**Ratio angiogenic genes**	1	7	8

**Limma cDNAs**	7	577	581

**Limma genes**	4	195	196

**Limma angiogenic genes**	3	62	62

**Combined cDNAs**	755	1671	2035

**Combined genes**	272	597	698

**Combined angiogenic genes**	74	139	171

This table conveys the numerical breakdown of results for the differential cDNA expression due to cold temperatures on the carp array. 171 were the total number of putative cold induced angiogenic genes.

### Initial cooling, fold ratio subtraction and microarray re-analysis

It is thought angiogenesis is switched on in the initial stages of cooling, before the animal has become acclimatised to a particular temperature. This has been shown in rats subjected to cooling to 4°C which led to a 2.7 fold increase in VEGF expression for 1 to 4 hours and returned to the basal level at 24 hours [8,9]. Therefore, the microarray data was analysed between the first and last time point at each target temperature.

First, ratios were calculated between each of the cooling time points *vs*. a control group (see methods). Analysing heart and muscle separately and combining the results gave 83 unambiguous cDNAs with a ratio subtraction > = 2 and this represented 30 non-redundant genes. 8 of these genes were predicted angiogenic, see table 3 and additional file 12, column e for both heart and muscle sheets.

To further discover genes switched on at initial cooling, microarray analysis using Limma from the BioConductor R package [68] was used to fit a linear model to the arrays (see methods). An adjusted p-value < = 0.05 was employed as a significance threshold. 7 and 577 unambiguous cDNAs were found to be up-regulated in heart and skeletal muscle respectively (table 3 and additional file 12; columns f and g, both heart and muscle sheets). Combined, this represented 196 non-redundant genes, of which 62 were predicted to be angiogenic (table 3 and additional file 12; columns f and g, both heart and muscle sheets).

### Differential gene expression on initial cooling: overlap with ratio subtraction and Limma analyses

Both these methods are seeking to find genes up-regulated at the first time point on immediate cooling of fish vs. fish that have acclimatised to the temperature. The results should corroborate each other. Dealing the heart tissue first, there were 7 cDNAs that reached a p-value < = 0.5 and 3 cDNAs that had a ratio subtraction > = 2 (see additional file 12, sheet 12a). These cDNAs did not overlap but the ratio subtraction results, though not significant, for the Limma cDNAs were positive. So they concurred but not with significance for the ratio subtraction.

Muscle, in contrast, gave better corroboration between ratio and Limma analyses. There were 577 cDNAs with Limma adjusted p-value < = 0.05. There were 82 total ratio subtractions that reached significance (see additional file 12, sheet 12b). Of these, 47 agreed with significant Limma results. The results were encouraging. In general, the number of genes predicted by a method are always a balance between false positives and false negatives. In this case 47 cDNAs are in common in total between the "ratio" and the "Limma" methods for muscle. However, differences between the two methods are expected as the first is based on a hard cut-off on the fold change, the second is based on a conservative cut-off on a p-value after multiple test correction (so it accounts for the variation, not only for the mean value; and also for multiple tests). The reason for using the different methods is: if 3 biological replicates were present than the second would be the ideal, however, here only different temperatures were used as replicates and as such, there were no real biological replicates. Therefore, the comparison between the methods might be more informative. In the results genes that are predicted by 1 method and not contradicted by the others are used: this is equivalent to lowering the multiple test correction threshold in one method when results agree with the other.

### Total cold induced genes

2035 cDNA sequences were unambiguously up-regulated for all three methods, representing 698 genes and 171 of these were identified as angiogenesis-related genes (table 3 and additional file 12).

### Summary of analyses

**Figure 4:**
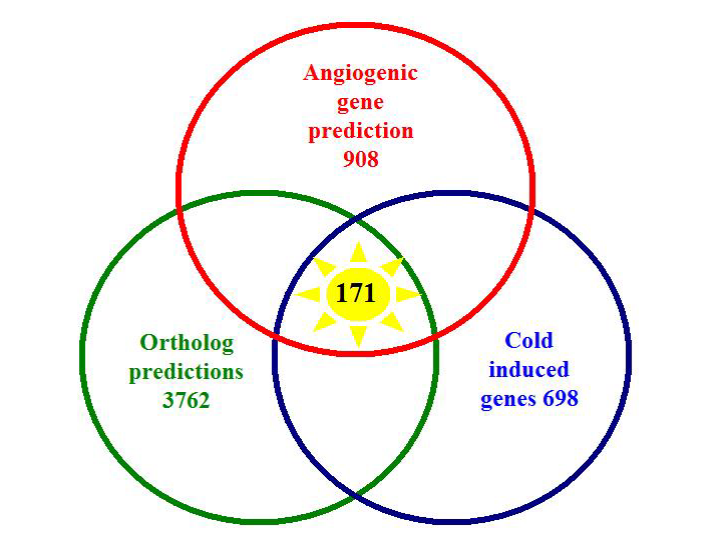
**Venn diagram summary**. The Venn diagram summarises the results from the different searches and analyses. 171 cold induced angiogenesis genes were predicted in this study.

The combined results of this analysis are summarised in figure 4 and are as follows: From the 16,650 contigs searched, 8,726 were successfully assigned an ortholog, which amounted to 3,762 non-redundant gene orthologs. 908 of the orthologs were predicted to be angiogenesis genes and 698 of the predicted orthologs were found to be induced by cold temperature. Finally, cross-referencing these genes found 171 cold induced angiogenic genes (additional file 13).

### Biologically relevant genes

The aim of this study was to develop a bioinformatic method of predicting genes of biological interest in a non-model species. Although many putative angiogenesis genes were found, some key angiogenic genes were not present on the carp array. For instance, vascular endothelial growth factor (VEGF) is a major activator of angiogenesis [69] and it would have been interesting to see the effect of cold exposure on this gene. Nevertheless, table 4 displays 12 out of the 171 angiogenesis-related genes up-regulated by low temperature. On investigation of these genes (highlighted on table 4 and below), there is encouraging evidence that these methods were successful.

**Table 4:** A selection of cold induced angiogenic genes predicted in this work

**Gene**	**Accession**	**Gene product**
APOE	NP_000032	apolipoprotein E

FN1	NP_997647	fibronectin 1

**GRN**	NP_002078	**granulin**

HMGB1	NP_002119	high-mobility group box 1

HSPG2	NP_005520	heparan sulfate proteoglycan 2

ITGAL	NP_002200	integrin, alpha L (antigen CD11A (p180), lymphocyte function-associated antigen 1; alpha polypeptide)

**MAPK1**	NP_002736	**mitogen-activated protein kinase 1**

**MMP9**	NP_004985	**matrix metallopeptidase 9 (gelatinase B, 92 kDa gelatinase, 92 kDa type IV collagenase)**

**NCL**	NP_005372	**nucleolin**

PLAUR	NP_002650	plasminogen activator, urokinase receptor

**RHOA**	NP_001655	**ras homolog gene family, member A**

SOD2	NP_001019636	superoxide dismutase 2, mitochondrial

From the 135 putative angiogenic genes up regulated due to cold temperatures, 12 genes considered biologically interesting in terms of angiogenesis are listed. The four highlighted in bold are reviewed in the text.

### Mitogen-activated protein kinase 1 (MAPK1)

MAPK1 is part of the MAPK-signalling pathway, which is the interface to a myriad of cellular processes like differentiation, proliferation and development [70-72]. It is also activated by two upstream kinases RAF and MEK, which are themselves activated by growth factor receptors [72]. Upon activation, MAPK1 is phosphorylated and translocates to the nucleus where it phosphorylates targets such as c-Fos, c-Jun, c-Myc and Tal-1 and initiates transcription of many genes [72]. During angiogenesis MAPK1 is activated in ECs and induces EC proliferation, critical to the establishment of new vessels [73], abrogates apoptosis and promotes the transcription of VEGF, itself a key activator of angiogenesis [74]. It is possible this is done *via *the hypoxia-inducible factor 1 alpha (HIF1A) gene as MAPK1 can activate HIF1A and this transcription factor is known to mediate VEGF expression [71,74]. The involvement of HIF1A in cold-induced angiogenesis fits well as reduced oxygen diffusion and increased blood viscosity can lead to tissue hypoxia.

Interestingly, the MAPK-pathway regulates expression of MMP9 [75,76] which also was predicted as a cold-induced angiogenic gene in this study. This finding is supported by the fact that inhibition of MEK, an upstream activator of MAPK1, curtails the expression of MMP9 [75] and that the MAPK-pathway induces transcription of MMP9 *via *the upstream promoter site AP-1 [77].

### Matrix metallopeptidase 9 (MMP9)

MMP9 is a member of a family of zinc containing endopeptidases (type IV collagenase) and plays a major role in angiogenesis [78,79]. MMP9 is synthesised as an inactive zymogen (pro-MMP9) that is activated by proteolysis [80]. During angiogenesis, MMP9 is secreted by endothelial cells to degrade the extracellular matrix, thus allowing ECs to migrate and form new blood vessels [81]. Coupled to this are the facts that MMP9 inhibition significantly decreased cerebral EC migration and proliferation [82] and that a positive correlation with micro vessel density and MMP9 expression exists [83]. MMP9 is also known to release VEGF from extracellular matrix (ECM) stores [84,85].

### Ras homolog gene family, member A (RHOA)

Another cold-induced angiogenic gene that was predicted in this study was RHOA, which is also known to induce MMP9 expression [86]. RHOA is a member of the RHO-family GTPases which are part of the RAS superfamily [87]. It has been shown that dominant-negative RHOA in ECs impairs tube formation and sprouting *in vitro *[88]. This is replicated *in vivo *where dominant-negative N19RhoA impairs vessel assembly and dominant-active V14RhoA stimulates ECs to form vessels [89]. RHOA has also been shown to induce cytoskeleton re-organisation, enhance migration and increase angiogenic capacity [90]. VEGF increases RHOA activity by enhancing its recruitment to the membrane and mediating the RhoA/Rho pathway during angiogenesis [91]. Over expression of dominant-active RhoA leads to increased tyrosine phosphorylation of VEGFR2, which is the key inducer of angiogenesis [92]. Again, related to hypoxia and low oxygen diffusion at low temperatures, RHOA is up-regulated in hypoxia and has been shown to be required for the accumulation of HIF1A, which induces VEGF expression [93].

### Granulin (GRN)

Some of the cold-induced genes have not been widely studied as angiogenic factors but have nevertheless been implicated in the process. These include granulin and nucleolin. Granulin is not a direct acting angiogenic factor but has been shown to stimulate VEGF expression in breast carcinoma cells [94]. Granulin has also been shown by yeast two hybrid assays to bind the HIV Tat protein that is actively secreted by HIV infected cells and acts as an EC growth and angiogenic factor [95]. In addition, granulin has been shown to be expressed within proliferating ovarian carcinomas blood vessels and interact with perlecan. Therefore, it is thought to regulate tumour angiogenesis and influence cancer growth [96]. Finally, an anti-granulin antibody has been shown to inhibit tumour angiogenesis in human hepatomas implanted into athymic mice [97].

### Nucleolin (NCL)

Nucleolin is one of the major proteins of the nucleolus but is also expressed on the cell surface where it binds a variety of ligands involved in several cell processes. Expression of nucleolin is particularly high on the surface of ECs in angiogenic blood vessels [98]. Inhibition of nucleolin in ECs prevents capillary tube formation and retards EC migration [99]. It is of interest that MMP9, also a predicted cold-responsive angiogenic gene in this study, is associated with nucleolin in angiogenesis. Mimicking hypoxia led to a 3-fold increase in MMP9 protein levels, due to enhanced translational efficiency caused by nucleolin binding to the 3' UTR of MMP9 [100]. These data showed that MMP9 expression during angiogenesis can be post-transcriptionally regulated by nucleolin. Lastly, antibodies to nucleolin have been shown to suppress tumour growth and angiogenesis [101].

## Conclusion

We provide evidence in this study that genes of biological interest in a non-model species, without a sequenced genome, can be discovered by combining re-analysis with a fast and efficient method of finding orthologs between fish and mammals. With the advent of next generation sequencing we envisage this approach will be useful to researchers doing similar studies in other organisms or for other biological processes. Programs using the methods from this work can be downloaded from  and can be used to run these analyses locally.

## Methods

### Bioinformatics analyses - sequence collection and contigs assembly

On the 23rd March 2007 the total number of cDNA sequences represented in the CarpBASE 3.0 [102,103] database publicly available for download was 17,825. Additional sequences from other sources were found using Entrez [104] to search Genbank (release 158, February 2007) for all carp ESTs and mRNAs. A total of 19,995 EST sequences were collected and clustered using the CAP3 [27] (Version Date: 04/15/05) software to create overlapping contigs of the same carp genes. All the default settings were employed except for a stringent overlap percent identity cut-off of 98%. Combining the singleton EST counts with the clustered contigs gave a total 16,650 sequences for use in ortholog searches. All the cDNA accession numbers are in additional file 1 (note: AJ577601 was removed at the author's request) and a key to which sequences were contained in the contiguous sequences is in additional file 2.

### Unbiased selection for evaluation of results

A systematic method of selecting results to analyse was required in this study to counteract any cherry picking bias. This was achieved by choosing random numbers from the RANDOM.ORG website [105], which generates random numbers based on atmospheric noise. These numbers determined the particular results that were looked at, and in most cases, 10 random results were chosen.

### Ortholog identification RBH, SRBH and CSRBH

The carp contigs were BLASTX searched with the stand alone NCBI BLAST [106,107] in all six frames against the human Refseq protein database [108]. The best hit was recorded and assigned as a putative ortholog. To increase the quality of the data, a reciprocal TBLASTN was performed which took the human ortholog protein and searched back against the carp contigs sequences. This was called the reciprocal best hit (RBH) [23,29,109] and was regarded as successful if the contig found in the RBH search represented the same human protein queried (figure 1, route 1).

The default BLAST algorithm uses a heuristic approach where it searches for small words (sequence regions) in the query and the subject that are exactly the same. It then attempts to extend out the matching words until a score threshold is reached to produce longer alignments. The Smith-Waterman algorithm [110], on the other hand, uses a dynamic programming method to produce an optimal local alignment [111,112]. This can be utilised in BLAST with -s T [113] options for BLASTP and TBLASTN. Different BLAST filtering performance were tested (-F F, -F "m S", -F T and -s T) on eukaryotic data to compare those results of prokaryotes [29]. All BLAST searches employed an e-value of 1e^-5 ^as done by Woods et al. 2005 [45] All orthologs that failed this step were removed.

A Stepped Reciprocal Best Hit (SRBH) was a two-stepped process where an RBH between carp and zebrafish was first performed and then for the successes, a second RBH was carried out between zebrafish and human. Only those sequences successful for both RBH searches were assigned orthologs (figure 1, route 2).

Finally, a Conditional Stepped Reciprocal Best Hit (CSRBH) approach was performed using a Perl program to post-process BLAST results. Carp sequences were BLAST searched against both human and zebrafish Refseq proteins and the best hit determined the route to ortholog identification (figure 1).

### Angiogenic genes

Four different methods were used to identify which genes are, or potentially are, angiogenic:

1) The in-house method involved collecting genes based on the authors own angiogenesis research, combined with genes on the SABiosciences [52] commercial angiogenic array and those listed in the literature [51].

2) Gene data was downloaded from Genbank [114] and Perl programs, in conjunction with PubMed [53], were used to find article abstracts that contained one or more of the following angiogenic keywords: angiogenic, angiogenesis, neovascularization, neovascularisation, vasculogenesis, hypoxia, endoth and VEGF.

3) Similarly, Gene Ontology data was downloaded from Genbank [114] and Perl programs were used to search the Gene Ontology process data for each protein. The same keywords were used as in the previous method.

4) Any genes differentially or specifically expressed in ECs are potential angiogenic genes. Therefore, an *in-silico *predicted endothelial transcriptome gene list [15,16] was compared with the genes in this data set to find those endothelial up regulated or specific. These genes that had a False Discovery Rate (FDR) qvalue of < = 0.01 were classed as endothelial.

### Cold response genes: persistently up-regulated genes

Cold response genes were found mining the microarray data from Gracey et al. 2004 [26]. Differentially expressed cDNAs for the heart and muscle were taken directly from the data without any processing. These were the first list of cold-induced genes.

Then a second list was generated on the hypothesis that angiogenesis is switched on at initial cooling and switched off after some time. Therefore, two further strategies were employed.

### Cold response genes: ratio subtraction

Gracey et al. 2004 [26] harvested RNA and ran microarrays for several time points at each drop in temperature (see their figure 1). As angiogenesis is hypothesised to be switched on at initial cooling, subtracting gene expression ratio on the 1^st ^day of cooling *vs*. the expression ratio on the last day will show which genes showed highest expression on initial cooling. This can be expressed as a simple equation:

A = Average gene expression fluorescence at 30°C, the control temperature

B = Fluorescence intensity at day 1 of cooled temperature e.g. 17°C at day 1

C = Ratio of fluorescence at day 1 *vs*. the control i.e. B/A

D = Fluorescence intensity at last day of cooled temperature e.g. 17°C at day 4

E = Ratio of fluorescence at last day *vs*. the control i.e. D/A

R = ratio subtraction = C - E

The higher value of R means gene expression was higher on the first day as compared to the last day.

### Cold-response genes: microarray methods

Pre-processed and normalised gene expression data were obtained from the study [26]. Analysis was carried out using Limma from BioConductor R package [68], a general linear model approach that uses an empirical Bayesian smoothing [115] method to gain power when a large number of predictors (i.e. probes) are present with a small number of cases (i.e. arrays). Contrasts were employed within Limma to detect probes for which the change in expression between the first day of cooling and the control was significantly different to the change in expression between the last time points and the control. The Benjamini and Hochberg method [116] was used to correct for multiple testing. Amongst significant probes, different patterns were observed. However, angiogenesis-related genes are predicted to be up-regulated in the initial stages of cooling, before the animal has become acclimatised to a particular temperature; therefore, genes that were up-regulated at the 1st time point but showed no differential regulation at the last day of cooling were selected from this list. An adjusted p-value < = 0.05 was used as a cut-off.

## Comparison with Homologene

Homologene orthologous group data was downloaded from the Homologene ftp site at the NCBI [117]. Then a Perl program compared the gene symbol assignments for human and zebrafish between the two data sources. additional file 6 contains the results.

### Programs for running ortholog searches

See  for programs enabling high-throughput ortholog assignment for incomplete nucleotide sequences. Please contact the first author for guidance as some customisation maybe needed depending on species.

## Abbreviations

Carp: *Cyprinus carpio*; RBH: Reciprocal Best Hit; SRBH: Stepped Reciprocal Best Hit; CSRBH: Conditional Stepped Reciprocal Best Hit; BLAST: Basic Local Alignment Search Tool; BLAT: BLAST Like Alignment Tool; cDNA: complementary DNA; DDD: Digital Differential Display; EST: Expressed Sequence Tag; VEGF: Vascular Endothelial Growth Factor; EC: Endothelial Cell; GEO: Gene Expression Omnibus; SAGE: Serial Analysis of Gene Expression; SW: Smith-Waterman; ECM: extracellular matrix; CGAP: Cancer Genome Anatomy Project; NCBI: National Center for Biotechnology Information; EBI: European Bioinformatics Institute

## Authors' contributions

JH performed the bioinformatic analyses and co-wrote the paper. FB performed the re-analysis of the microarray data. HV aided in interpretation of the data and helped write the paper. SE and RB conceived the overall project, helped with the interpretation of the data and writing of the paper. All authors read and approved the final manuscript.

## Supplementary Material

Additional file 1 **This file contains the accession numbers for all the 19,995 sequences downloaded from Genbank and used in these analyses**. Click here for file

Additional file 2 **This file contains a key to which cDNA accession numbers have been clustered into contiguous sequences using CAP3.** Click here for file

Additional file 3 **This file shows the alignment of sequences that give evidence that Smith-Waterman alignments did not always outperform the normal BLAST algorithm in these tests.** Click here for file

Additional file 4 **BLAST data, though not definitive, gives some evidence for the occurrence of a genome duplication event in zebrafish. It shows that two zebrafish set genes best match the same human SET gene.** Click here for file

Additional file 5 **Evidence is presented in this file to show that the SRBH method outperforms Homologene for some genes and that phylogenetic analysis does not always assign the correct ortholog.** Click here for file

Additional file 6 **A comparison was made between the SRBH method and Homologene Orthologous group database of the zebrafish to human ortholog assignments by SRBH. 85% of assignments agreed between the two data sources.** Click here for file

Additional file 7 **10 examples were picked at randomly from the Homologene comparison that did not agree with the RBH results.** Results found that in 80% of the cases, the RBH method looked like the correct assignment. Click here for file

Additional file 8 **Data is presented in this file that shows the failed SRBH steps of EST EC394432 ortholog assignment.** Click here for file

Additional file 9 **This file presents further evidence to use a CSRBH as against a SRBH using the carp EST AU052068 as an example.** Click here for file

Additional file 10 **From the 16,665 carp contiguous sequences searched using the Conditional Stepped Reciprocal Best Hit (CSRBH) method, 8,726 sequences were successfully assigned a human ortholog.** This table displays the ortholog assignments and contains 3,762 non-redundant human genes. Click here for file

Additional file 11 **From the 4 methods used to predict angiogenesis genes, this table lists the 908 genes that were predicted as angiogenic.** Click here for file

Additional file 12 **This file contains a list of cDNAs that were found to be up-regulated in the Gracey et al. 2004 data set from all three different re-analysis methods.** Click here for file

Additional file 13 **This table contains the 171 predicted cold induced angiogenesis genes.** Click here for file
