# Supplementary material for: A new procedure for determining the genetic basis of a physiological process in a non-model species, illustrated by cold induced angiogenesis in the carp
Source: BMC Genomics. 2009 Oct 23;10:490. doi: 10.1186/1471-2164-10-490 (PMC2771047; doi:10.1186/1471-2164-10-490)
Supplement: Additional file 7 — 10 examples were picked at randomly from the Homologene comparison that did not agree with the RBH results. Results found that in 80% of the cases, the RBH method looked like the correct assignment. [file 1471-2164-10-490-S7.DOC]

## Additional file 7:

10 examples were picked at randomly from the Homologene comparison that did not agree with the RBH results. Results found that in 80% of the cases, the RBH method looked like the correct assignment.

Example 1:

From a RBH analysis the Contig1176 matched zebrafish NP_775369 and human NP_066357 (RPL36A) was predicted as its functional ortholog. Aligning these proteins produced gave an alignment of 98% identity over the full length of both proteins. In Homologene, these genes were put into two different orthologous groups; the zebrafish protein NP_775369 in group 776 and the human protein NP_066357 into group 122207 (original results was in group 107247).

Example 2:

The cDNAs CA968922, DW722743, CA968735 are successfully assigned the human gene HEBP2 using RBH. The zebrafish protein XP_001922019 and the human protein NP_055135 are put into two orthologous groups at homologene, 120680 and 8634 respectively. They both contain a single SOUL (pfam04832) domain. However, the human gene hits another zebrafish protein with high similarity, NP_956492, therefore, it is predicted the two zebrafish genes arise from a genome duplication or other gene duplication and this is catered for and not missed in the CSRBH approach whereas it they are put into two different orthologous groups in Homologene.

Example 3:

Contig1321; the human protein NP_036200 RBH success was to zebrafish protein XP_695637. However, Homologene placed them in different orthologous groups (32142 and 121459) even though they contain the same transcription factor domain.

Example 4:

Contig1474; the human protein NP_055063 is the gene RBX1, in zebrafish the same gene rbx1has the protein XP_693916. Both these genes match with high similarity and with the same kind of domain. However, Homologene places them in different orthologous groups, 105492 (Human) and 6872 (Zebrafish).

Example 5:

The three cDNAs CF660398, CA968407 and CA964538 were all assigned the human gene EGLN1 and the successful RBH from zebrafish matched NP_001002595 egl nine homolog 1. Homologene puts the two genes, both called EGL nine 1 into different orthologous groups, however they are given the same gene symbol. This gene could be due to a gene duplication event in zebrafish or genome duplication as one domain is present and the other is not.

Example 6:

Human protein NP_003286 and zebrafish protein XP_001334958 are placed in different orthologous groups (105592 and 55730) but they have the same domain and nothing else has a better sequence similarity.

Example 7:

Contig109; the protein in human NP_005548 found the zebrafish protein NP_001003445. Again, in Homologene, they were placed in different homologous groups (21145 363).

Example 8:

AU183426; Homologene has correctly put these genes in different orthologous groups based on domain structure. The human protein is missing one Pyridine nucleotide-disulphide domain. The human protein NP_001087240 was in orthologous group 55733 and the zebrafish protein NP_898895 in group 60033.

Example 9:

CA964824; the human protein NP_075385 was assigned a different orthologous group to zebrafish protein NP_998388 (Orthologous groups 40748 and 120328). They have no recognised functional domains but they have very similar gene symbols. It is likely the RBH method correctly assigned them functional homologs.

Example 10:

EC392776; both the human protein NP_001074318 and zebrafish protein XP_001334530 contain a zinc finger domain and they match with high similarity. They are placed in different orthologous groups in Homologene, groups 36864 and 122492.
